# Nomogram incorporating ultrasonic markers of endometrial receptivity to determine the embryo-endometrial synchrony after *in vitro* fertilization

**DOI:** 10.3389/fendo.2022.973306

**Published:** 2022-12-16

**Authors:** Qi He, Ying Zhou, Weiqin Zhou, Caiping Mao, Qian Kang, Yanping Pan, Nan Wang, Yanyu Zhong, Zhansheng Pan

**Affiliations:** ^1^ Reproductive Medicine Centre, The First Affiliated Hospital of Soochow University, Suzhou, China; ^2^ Department of General Surgery, The First Affiliated Hospital of Soochow University, Suzhou, China

**Keywords:** nomogram, *in vitro* fertilization, embryo transfers, endometrial receptivity, embryo-endometrial synchrony, window of implantation

## Abstract

**Background:**

A successful pregnancy using *in vitro* fertilization and embryo transfer (IVF-ET) requires a receptive endometrium, good-quality embryos, and a synchronized embryo-endometrial dialogue. Although embryo quality and endometrial receptivity (ER) have been fully assessed to exclude substandard conditions, the probability of successful ET is relatively low. Currently, embryo-endometrial synchrony is considered to be a possible explanation, because delayed, advanced, or narrowed window of implantation (WOI) may lead to ET failure.

**Objective:**

This study aims to establish a nomogram incorporating a series of ultrasonic ER markers on the day before implantation to assess the embryo-endometrial synchrony, which may contribute to the improvement of clinical pregnancy outcomes.

**Methods:**

Totally 583 women with 1135 complete IVF cycles were retrospectively analyzed. Among them, 357 women with 698 cycles and 226 women with 437 cycles were assigned to the training and validation cohorts, respectively. Ultrasonic ER markers obtained on the day before implantation were collected for analyses. In the training cohort, the screened correlates of clinical pregnancy failure were utilized to develop a nomogram for determining whether an infertile woman is suitable for the ET next day. This model was validated both in the training and validation cohorts.

**Results:**

Spiral artery (SA) resistance index (RI), vascularisation index (VI), and flow index (FI) were independently associated with the ET failure (all *P* < 0.05). They were served as the components of the developed nomogram to visualize the likelihood of implantation failure in IVF-ET. This model was validated to present good discrimination and calibration, and obtained clinical net benefits both in the training and validation cohorts.

**Conclusion:**

We developed a nomogram that included SA-RI, VI, and FI on the day before implantation. It may assist physicians to identify patients with displaced WOI, thus avoiding meaningless ET prior to implantation.

## Introduction

*In vitro* fertilization and embryo transfer (IVF-ET) has been recognized as an effective assisted reproduction technology (ART) for the treatment of infertility (1, 2). A successful pregnancy requires a receptive endometrium, good-quality embryos, and a synchronized embryo-endometrial dialogue (3). Despite the in-depth understanding of embryo quality and endometrial receptivity (ER), the probability of embryo implantation is only about 30% (4). It has been reported that more than half of good-quality embryos fail to implant in the receptive endometrium, indicating that suboptimal embryo-endometrial synchrony may play a crucial role in implantation failures (5–7).

The first-line assessment of ER is transvaginal sonography (TVS), and endometrial thickness (EMT), volume, pattern, and blood flow are the typical use of ER markers (6, 8). However, ER is a complicated process that allows embryonic attachment, invasion, and development. The endometrium is unique in its ability to prevent embryos from implanting, except during the window of implantation (WOI) (9). The optimal WOI has been determined to be not consistent among all women (10). Implantation failure is more common in women who have WOI displacement, which may delay, advance, or narrow the WOI due to unknown contributing variables that disturb the ER (9, 11–13). Considering the high treatment costs and the potential effects on female emotions, more accurate determination of the optimal WOI is needed for improving clinical pregnancy outcomes (14, 15).

Prediction models in IVF, which quantify a risk by combining several prognostic factors, are regarded to be effective in providing adequate advice on the chances of conceiving using IVF-ET (16). However, to our knowledge, there are few reports for accurate identification of the optimal WOI by establishing a nomogram that incorporates ultrasonic ER markers. With this background, the purpose of the present study is to establish a nomogram incorporating a series of ultrasonic ER markers to assess whether a patient is at the optimal WOI, so as to improve the clinical pregnancy outcomes after IVF-ET.

## Materials and methods

This study was approved by the institutional review board of The First Affiliated Hospital of Soochow University (2020-137), and informed consents were obtained from all participants. It was complied with the principles stated in the Declaration of Helsinki.

### Study participants

From January 2019 to February 2022, participating in this retrospective study were 785 consecutively enrolled infertile women who underwent IVF-ET at the Reproductive Medicine Center of The First Affiliated Hospital of Soochow University. Their demographics and medical histories were collected at the first visit.

The following were the inclusion criteria for infertile women to reduce the impact of confounding variables on this study: 1) age 20-39 years; 2) at least one good-quality embryo transferred in each cycle; 3) normal ovarian reserve; 4) no hormonal medication in the past 2 months. Those patients were excluded based on the following criteria: 1) endometrial diseases (such as intrauterine adhesions, endometrial polyps, or endometritis, etc.); 2) uterine cavity occupation diseases (such as intramural hysteromyomas, adenomyomas, or submucous myomas, etc.); 3) hydrosalpinx; 4) repeated implantation failure (17); 5) autoimmune diseases or prethrombotic state; 6) serious endocrine system diseases.

**Figure 1:**
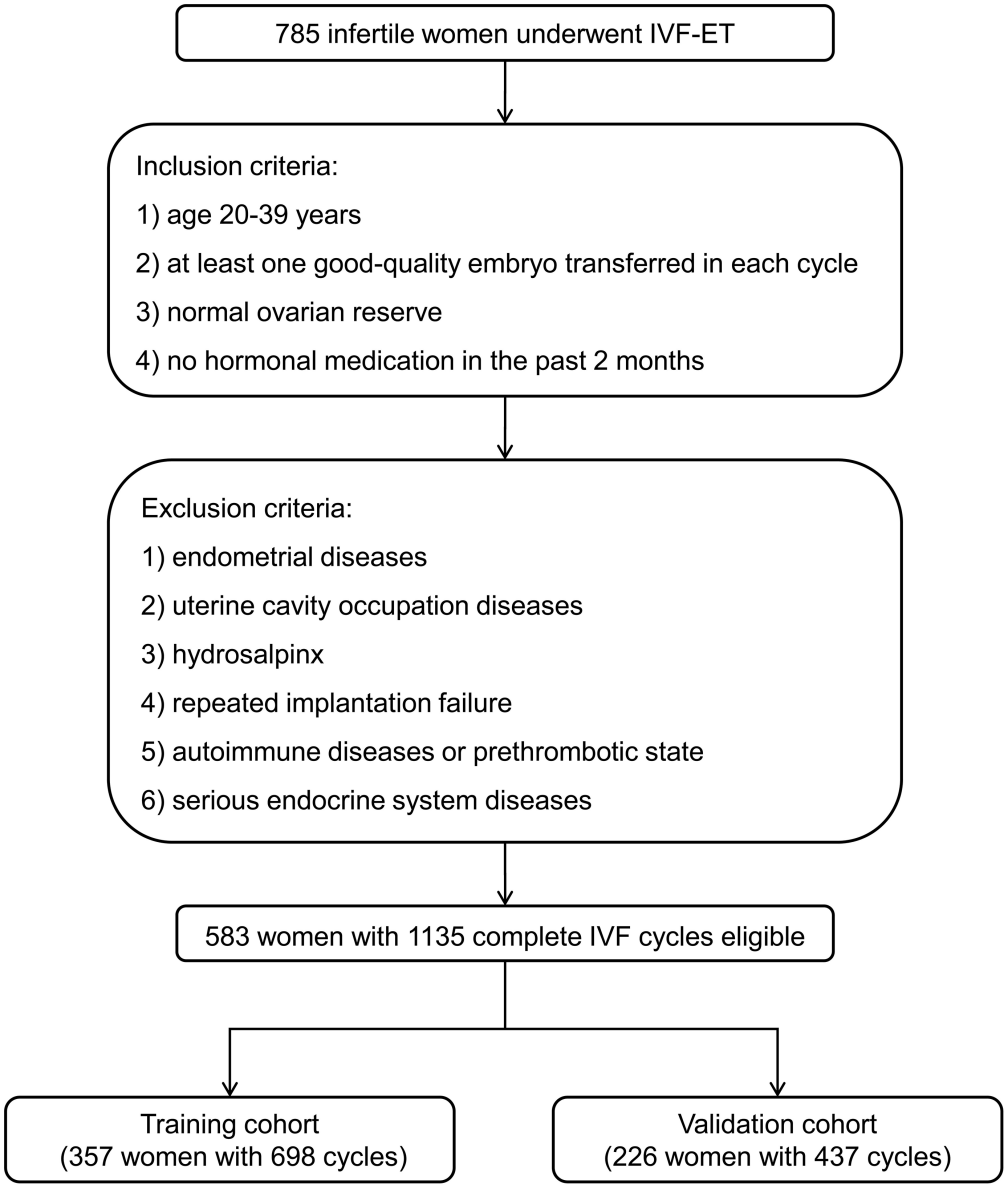
Flowchart of patient selection for developing the training and validation cohorts. IVF-ET, *in vitro* fertilization and embryo transfer.

Thus, 583 women with 1135 complete IVF cycles were eligible for the analysis. In order to independently validate the established nomogram, women with the IVF cycles from January 2019 to January 2021 were included in a training cohort (357 women with 698 cycles) and those from February 2021 to February 2022 were assigned to a validation cohort (226 women with 437 cycles) (**Figure 1**).

### IVF-ET procedure

All infertile women received controlled ovarian hyperstimulation (COH), oocyte retrieval, and fertilization, followed by a planned transfer of 1-2 embryos, with at least one good-quality embryo (18). Briefly, 10,000 IU of human chorionic gonadotropin (hCG) were given until at least two or three follicles measured ≥ 18 mm to trigger ovulation. Recombinant follicle-stimulating hormone or human menopausal gonadotrophin was used based on the actual condition of different individuals. Oocyte retrieval was carried out 34–36 hours following the hCG injection. Fresh cycle was performed and 1-2 day-3 embryos were transferred only when patients presenting receptive endometrium. Luteal phase support was initiated after oocyte retrieval and lasted for 10 weeks of gestation if pregnant. Frozen cycle was chosen if fresh cycle is not suitable due to maternal or embryonic issues. All embryos were cryopreserved and no luteal-phase support was administered after oocyte retrieval. At a new menstrual cycle, endometrium preparation was performed with a natural cycle regimen or hormone replacement therapy according to the patient’s condition. When the endometrium becomes receptive, 1-2 frozen-thawed day-3 embryos or day-5 blastocysts were transferred.

### Ultrasonic examination

Ultrasonic examinations were performed using Voluson™ E8 (GE, Boston, MA, USA) with a RIC-9-D intracavity probe by two experienced sonographers. On the day before implantation, all infertile women were routinely examined following a standard protocol. Ultrasonic data obtained in this study included EMT, endometrial pattern [(types A (triple-line), B (isoechoic), and C (hyperechoic) endometria], and average blood flow parameters of uterine artery (UA) and spiral artery (SA) [pulsatility index (PI) and resistance index (RI)]. Endometrial volume, vascularisation index (VI), flow index (FI), and vascularisation flow index (VFI) were measured in three-dimensional mode.

### Nomogram development

Clinical pregnancy, which was the primary outcome of this study, was defined as the observation of a gestational sac with embryo bud under TVS 4 weeks after embryo implantation. In the training cohort, we investigated the variables related to pregnancy outcomes by comparing the demographics and medical histories of women with successful and failed pregnancies. We then selected individuals who received multiple ETs and finally had a successful pregnancy, and compared their ultrasonic ER markers at the first-time failure and the successful ET to explore the variables associated with WOI based on a matched pairs design. Then all initial predictors were introduced to a multivariate logistic regression in order to investigate the independent correlates of pregnancy failure. Finally, these independent variables were utilized to develop a nomogram for determining whether an infertile woman is suitable for the ET next day. The performance of the model was first assessed in the training cohort and then externally validated by fitting it to the validation cohort with the same parameter estimates.

### Statistical analysis

Normally distributed continuous variables, which were tested by the Kolmogorov-Smirnov test, were expressed as mean ± standard deviation, while those with skewed distribution were expressed as median (interquartile range). Categorical variables were expressed as number of cases and constituent ratio [n (%)]. The nomogram was established by incorporating the independent correlates of pregnancy failure and odds ratios (ORs), as well as 95% confidence intervals (CIs) calculated by the multivariate logistic analysis. Internal and external validations were applied to evaluate the performance of the nomogram. Bootstraps (1,000 times) analyses were implemented to counteract the possible overfitting deviation. The discrimination of the model was quantified by the area under the receiver operating characteristic (ROC) curve (AUC). The performance of calibration was assessed by a calibration curve accompanied by a Hosmer-Lemeshow (HL) test. A decision curve analysis (DCA) was applied to determine the clinical applicability of our model by assessing the net benefits at different threshold probabilities. All statistical analyses were carried out using SPSS software (Version 22.0), Medcalc (Version 22.0.22), and R package (Version 4.1.3).

## Results

### Participants characteristics

Of the 583 eligible participants with 1135 complete IVF cycles, 374 (64.2%) achieved a clinical pregnancy. Among them, 131 (35.0%) were successful at the first ET, 201 (53.8%) at the second ET, and 42 (11.2%) at the third ET. Of the 209 women with final pregnancy failure, 112 (53.6%) failed three times, and 97 (46.4%) failed two times. **Supplementary Table S1** gives an overview of the demographics and medical history of women in the training and validation cohorts. No significant differences were observed between the two cohorts (all *P* > 0.05).

### Variables associated with ET failure

In the training cohort, the comparisons of the demographics and medical histories between women with successful and failed pregnancies are listed in **Table 1**. No variable was revealed to be related to the successful ET (all *P* > 0.05). For the participants who received multiple ETs and finally had a successful pregnancy (152 women in the training cohort), we compared their ultrasonic ER markers at the first-time failure and the successful ET (**Table 2**). It revealed that SA-PI, SA-RI, endometrial volume, VI, FI, and VFI were associated with the optimal WOI.

**Table 1:** Comparison of the demographics and medical histories between women with successful and failed pregnancies.

Variable		Successful pregnancy (n=237)	Failed pregnancy (n=120)	*P* value
Age, years		31 (29-34)	32 (30-34)	0.609^b^
BMI, kg/m^2^		23.1 (21.6-25.4)	22.7 (21.1-24.9)	0.612^b^
Duration of infertility, years		3 (1-4)	3 (2-5)	0.134^b^
Infertility, n(%)	Primary	119 (50.2%)	58 (48.3%)	0.737^a^
	Secondary	118 (49.8%)	62 (51.7%)	
Gravidity	0	119 (50.2%)	58 (48.3%)	0.922^a^
	1	66 (27.9%)	32 (26.7%)	
	2	35 (14.8%)	21 (17.5%)	
	≥3	17 (7.2%)	9 (7.5%)	
Parity	0	205 (86.5%)	105 (87.5%)	0.465^a^
	1	29 (12.2%)	15 (12.5%)	
	≥2	3 (1.3%)	0 (0%)	
History of abortion	No	148 (62.5%)	67 (55.8%)	0.228^a^
	Yes	89 (37.6%)	53 (44.2%)	
Surgical abortion	0	202 (85.2%)	96 (80.0%)	0.269^a^
	1	22 (9.3%)	12 (10.0%)	
	≥2	13 (5.5%)	12 (10.0%)	
Living children	0	216 (91.1%)	112 (93.3%)	0.473^a^
	≥1	21 (9.9%)	8 (6.7%)	

^a^for chi-square test, and ^b^for Mann-Whitney U test. BMI, body mass index.

**Table 2:** Comparison of the ultrasonic ER markers at the first-time ET failure and the successful ET in women from the training cohort.

Variable		Failed ET (n=152)	Successful ET (n=152)	*P* value
EMT, mm		10.23±1.64	10.84±1.52	0.383^c^
Endometrial pattern, n(%)	Type A	23 (15.3%)	21 (14.4%)	0.135^d^
	Type B	57 (38.0%)	61 (39.6%)	
	Type C	72 (46.7%)	70 (46.0%)	
SA-PI		0.91 (0.84-0.97)	0.88 (0.80-0.95)	0.036^e^
SA-RI		0.56 (0.53-0.62)	0.55 (0.51-0.59)	<0.001^e^
UA-PI		2.17 (1.82-2.47)	2.28 (1.86-2.67)	0.245^e^
UA-RI		0.56 (0.52-0.60)	0.55 (0.51-0.59)	0.627^e^
Endometrial volume, ml		4.84±1.17	5.22±1.24	0.016^c^
VI, %		2.82±0.78	3.25±0.89	<0.001^c^
FI		20.00±3.50	22.49±4.39	<0.001^c^
VFI		1.08±0.49	1.33±0.49	<0.001^c^

^c^for paired t-test, ^d^for McNemar’s test, and ^e^for Wilcoxon signed-rank test. ET, embryo transfer; BMI, body mass index; EMT, endometrial thickness; SA, spiral artery; UA, uterine artery; PI, pulsatility index; RI, resistance index; VI, vascularisation index; FI, flow index; VFI, vascularisation flow index.

### Independent correlates of implantation failure

**Figure 2:**
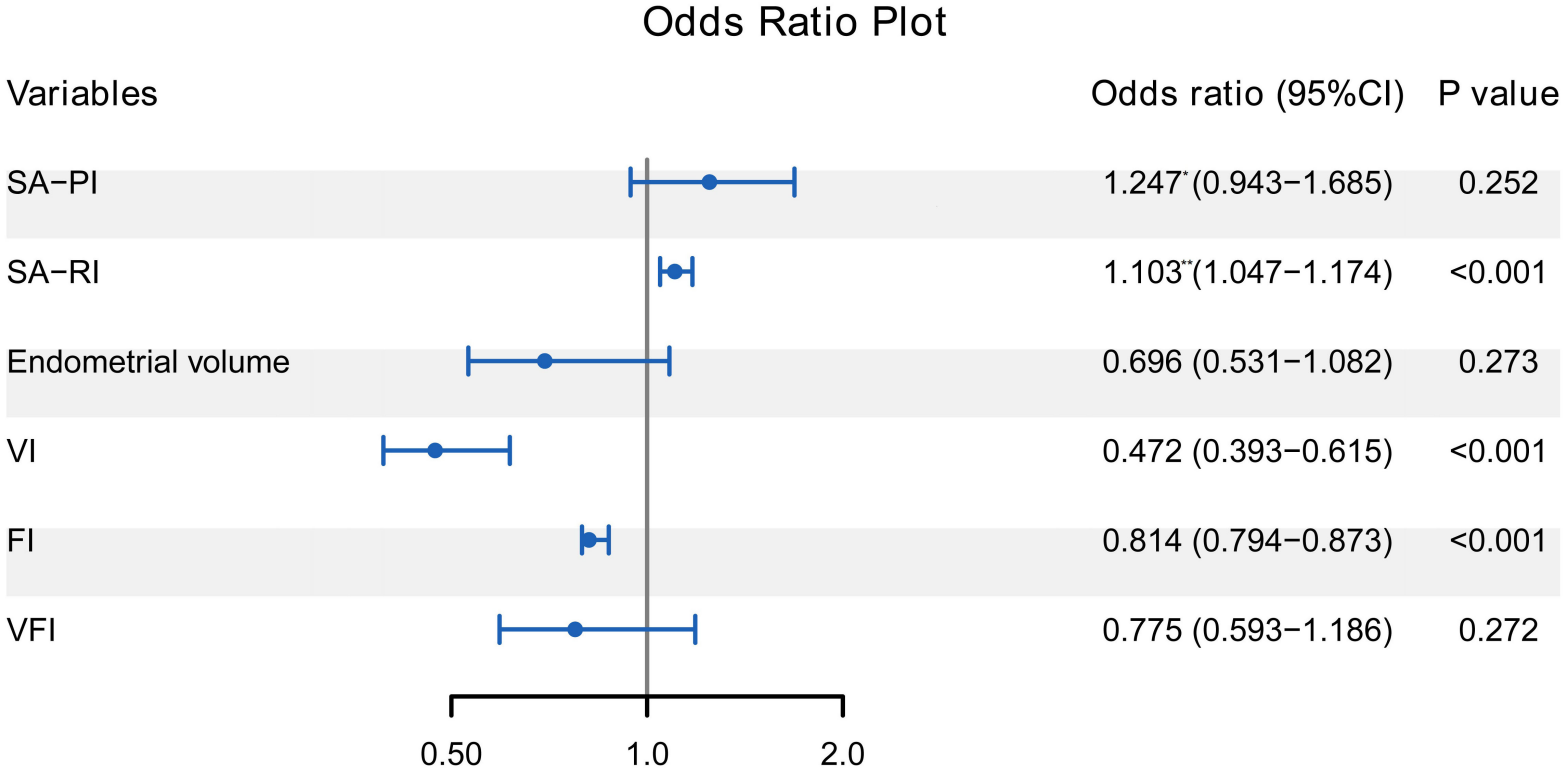
Forest plot of multivariate logistic regression analysis for the correlates of ET failure. SA-RI, VI, and FI were independently associated with ET failure (all *P* < 0.05). The odds ratio represented by * is the increased risk per 0.1 unit increase, and the ** represents the increased risk per 0.01 unit increase. ET, embryo transfer; SA, spiral artery; PI, pulsatility index; RI, resistance index; VI, vascularisation index; FI, flow index; VFI, vascularisation flow index.

The 6 variables further entered the multivariate logistic regression analysis to investigate the independent correlates of ET failure. **Figure 2** revealed that SA-RI, VI, and FI were independently associated with ET failure (all *P* < 0.05). Increasing SA-RI was more likely to lead to ET failure, with an OR of 1.103. Increased VI and FI helped minimize the risk of ET failure with ORs of 0.472 and 0.814, respectively.

### Model development based on the training cohort

**Figure 3:**
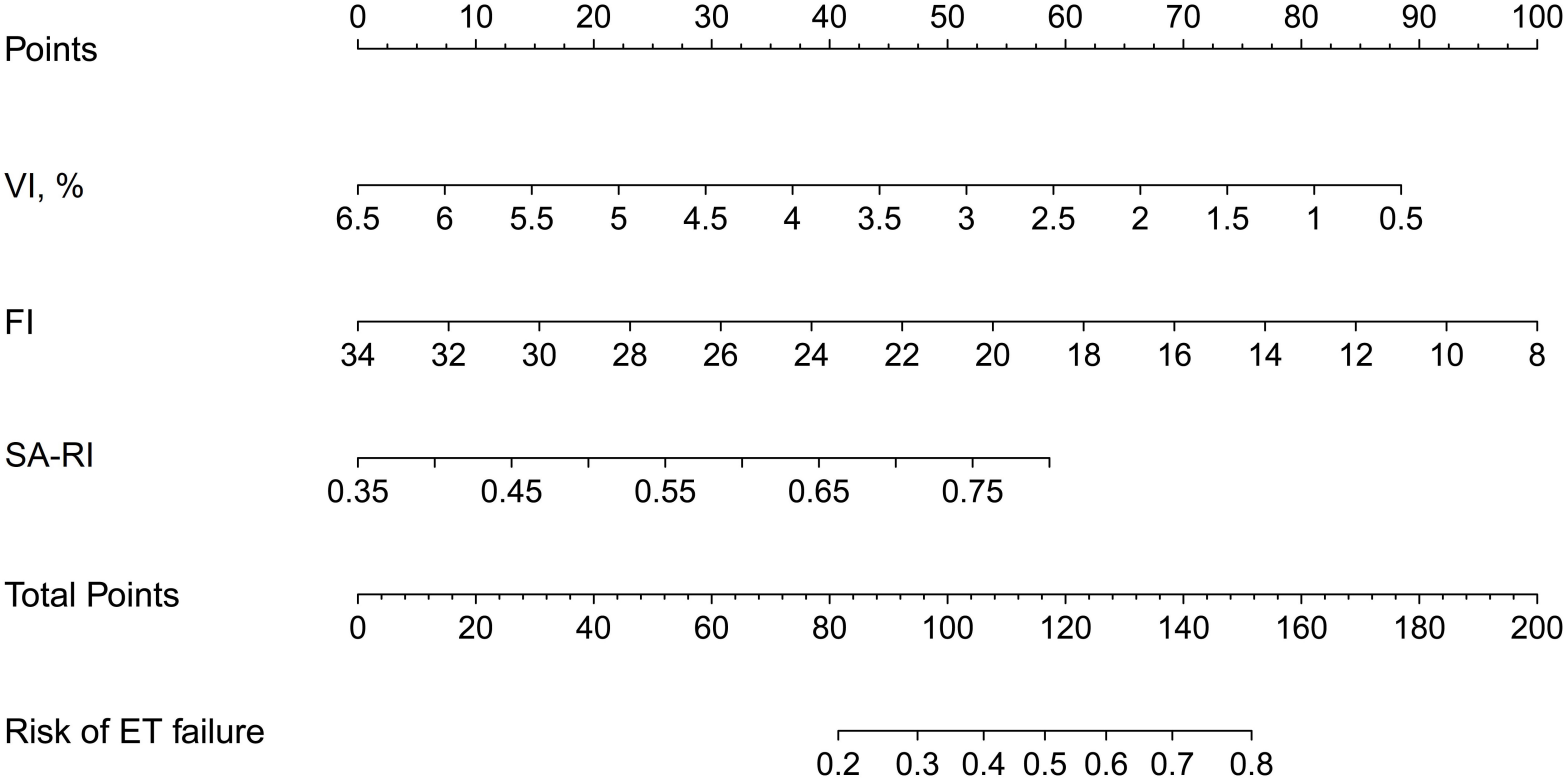
Nomogram for estimating the likelihood of implantation failure in IVF-ET. It is developed with SA-RI, VI, and FI with weights equal to the OR values. IVF-ET, *in vitro* fertilization and embryo transfer; SA, spiral artery; RI, resistance index; VI, vascularisation index; FI, flow index; OR, odds ratio.

A nomogram was constructed by combining the three selected variables to visualize the likelihood of implantation failure in IVF-ET. As indicated in **Figure 3**, the estimated probability of ET failure was derived by summing the points of each variable, with weights equal to the OR values. The sum included the total point and matched the risk on the bottom axis.

### Model validation

**Figure 4:**
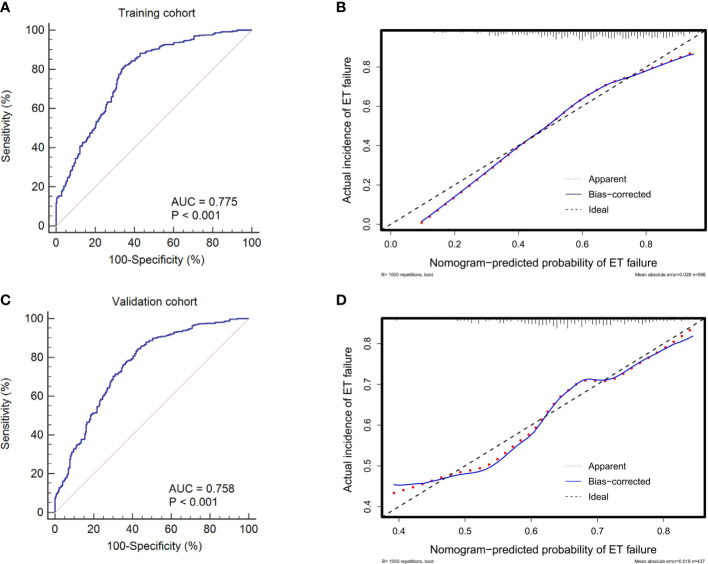
ROC curves and calibration plots of the training and validation cohorts to evaluate the discrimination and calibration of the nomogram. The AUCs of ROC curves are 0.775 **(A)** and 0.758 **(C)** in the training and validation cohorts, both indicating good discrimination. The calibration curves indicate that the predicted probability of ET failure match the actual incidence well both in the training **(B)** and validation cohorts **(D)**. ET, embryo transfer; ROC, receiver operating characteristic; AUC, area under the curve.

In the internal validations based on the training cohort, the model stability was verified after 1,000 bootstrapping to adjust the overfitting deviation. The discrimination and calibration of the nomogram were tested. As indicated in **Figure 4A**, the AUC of the ROC curve was 0.775 (95% CI: 0.742 to 0.805), which denoted a good performance in discrimination. The calibration curve (**Figure 4B**) and HL test revealed no significant difference between the predicted and actual probabilities of implantation failure (*χ^2^* = 10.375, *P* = 0.264), which suggested that the model was well-calibrated.

The external validations using the validation cohort were performed to test the usefulness of the nomogram. After the original model was applied to the validation cohort, the nomogram also showed good discrimination (AUC = 0.758, 95% CI: 0.725 to 0.790) (**Figure 4C**) and good calibration (**Figure 4D**) as assessed by the HL test (*χ^2^* = 9.682, *P* = 0.383).

### Clinical applicability of the nomogram

**Figure 5:**
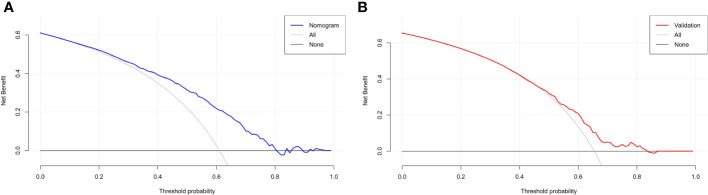
DCA based on the training and validation cohorts for evaluating the clinical utility of the nomogram. **(A)** reveals that in the training cohort, the nomogram yields clinical net benefits when the threshold probability is between 20% and 80%. **(B)** suggests that in the validation cohort, clinical net benefits are obtained when the threshold probability is between 45% and 80%. Both indicate good potential for clinical utility. DCA, decision curve analysis.

The DCA plot in the training cohort showed that if the threshold probability was between 20% and 80%, the use of the nomogram to predict the risk of ET failure generated a clinical net benefit (**Figure 5A**). The DCA plot in the validation cohort still showed net benefits although they were lower than the training cohort (**Figure 5B**). It yielded clinical net benefits when the threshold probability was between 45% and 80%, indicating a good potential for clinical utility.

## Discussion

Consistently, implantation failure in IVF-ET is a challenge with complex etiologies. Despite the comprehensive understanding of embryo quality and ER, most good embryos fail to transfer on the premise of eligible ER. Thus, WOI displacement, which interferes with the optimal embryo-endometrial synchrony, becomes the 3^rd^ potential reason of failed implantation. In this study, we developed and validated a nomogram to identify IVF cycles at risk of ET failure. This nomogram consisted of SA-RI, VI, and FI on the day before implantation, which were all independently associated with the ET failure. If further validated, it may determine whether a patient is at the optimal WOI prior to the ET day, although it is unable to assess whether the WOI is advanced or delayed. It is possible to stop the underperforming cycle for a new one if the patient is at risk of a WOI displacement, thus improving the clinical pregnancy outcome.

Asynchronous embryo-endometrial development, in addition to embryo quality and endometrial receptivity, has been shown to reduce implantation success. Successful implantation requires a synchronized development of a viable embryo and a receptive endometrium (11). However, many different events, such as COH as routinely utilized in IVF cycles to ensure multiple oocyte development, may disrupt embryo-endometrial synchrony (19, 20). Although improved embryo-endometrial synchrony relies on better assessment of endometrial development, it is unlikely to systematically investigate the optimal embryo-endometrial synchrony due to ethical reasons. In contrast to accurate assessments of embryo maturation, current endometrial development assessments mainly rely on non-invasive methods such as ultrasonography. However, it has been noted that well-studied ultrasonic parameters such as EMT do not accurately predict endometrial development (21). It appears that single ultrasonic markers are unlikely to accurately assess embryo-endometrial synchrony. Since IVF is physically and emotionally stressful, infertile women should be well informed about their chances of ET success prior to each cycle. Therefore, a nomogram prediction model may improve the accuracy of endometrial development assessment and reliably predict subsequent ET outcomes.

Although non-invasive assessments of endometrial development remain to be further investigated, our study revealed that a combination of SA-RI, VI, and FI on the day before implantation, help determine whether a patient was at the optimal WOI. In our investigation, SA-RI, VI and FI on the day before implantation were independently associated with the optimal WOI. It is well known that the branch arteries of the endometrial spiral arteries are widely recognized as the main nutrient source of the endometrium and are ideal predictors of endometrial receptivity (22). Several studies have demonstrated the effectiveness of ultrasound to assess endometrial receptivity by measuring endometrial blood flow (23–25). While our study found that on the day before implantation, elevated SA-RI, as well as decreased VI and FI, were also valuable in assessing embryo-endometrial synchrony.

A number of previous studies report prediction models for pregnancy after IVF-ET (16, 26, 27). Only a few of them have developed nomograms to predict clinical pregnancy based on acoustic parameters related to ER (28, 29). To our knowledge, this is the first study to determine whether a patient is at the optimal WOI for each IVF cycle by a nomogram. The established nomogram in this study was verified to show good discrimination and calibration, as well as significant clinical net benefits in predicting the risk of ET failure both in the training and validation cohorts. With this nomogram, IVF cycles with suboptimal WOI may be canceled prior to implantation until an IVF cycle with optimal WOI appears. For example, a 32-year-old woman, her VI, FI, and SA-RI measured the day before implantation was 2.9%, 19, and 0.5, respectively. The corresponding scores for these features in the nomogram were: 52 points for VI, 58 points for FI, and 20 points for SA-RI. Her total score was about 130, indicating that her risk of ET failure was about 64%. This cycle should be discontinued in order to choose a more appropriate cycle. If needed, the patient may need targeted therapy to improve endometrial perfusion in order to keep the risk below 50%.

Several limitations in this study should be acknowledged. Despite the relatively large sample size and the model verification in a validation cohort, it was a single-center retrospective study. Further validations based on other centers are necessary to remedy our model. In addition, selection bias was inevitable and some indicators with potential interference to WOI displacement were not included, such as smoking (30) and alcohol consumption (31). Therefore, multicenter prospective investigations are required in the future to strengthen the individualized decision-making through our model.

## Conclusion

In this study, we developed a nomogram that included SA-RI, VI, and FI on the day before implantation, for estimating the likelihood of ET failure in an IVF cycle. It may identify patients with displaced WOI, thus avoiding meaningless ET. This model may assist physicians to improve clinical pregnancy outcomes in IVF-ET by assessing embryo-endometrial synchrony prior to implantation.

## Data availability statement

The raw data supporting the conclusions of this article will be made available by the authors, without undue reservation.

## Ethics statement

The studies involving human participants were reviewed and approved by Ethical approval for the study was obtained from the ethics committee of The First Affiliated Hospital of Soochow University (2020-137). The patients/participants provided their written informed consent to participate in this study.

## Author contributions

Study design: QH, YiZ, and ZP. Data collection and analysis: QH, WZ, CM, QK, and YP. Supervision: YiZ and ZP. Statistics: QH, QK, YP, NW, and YaZ. Manuscript writing: QH, YiZ, WZ, and CM. Manuscript revision: QH, YiZ, and ZP. All authors contributed to the article and approved the submitted version.
