# Supplementary material for: Nomogram incorporating ultrasonic markers of endometrial receptivity to determine the embryo-endometrial synchrony after in vitro fertilization
Source: Front Endocrinol (Lausanne). 2022 Dec 16;13:973306. doi: 10.3389/fendo.2022.973306 (PMC9800505; doi:10.3389/fendo.2022.973306)
Supplement: Supplementary file 1 [file Table_1.docx]

**Supplementary**

**Table S1**. Demographics and medical histories of women in the training and validation cohorts

| Variable | | Training cohort (n=357) | Validation cohort (n=226) | *P* value |
| --- | --- | --- | --- | --- |
| Clinical pregnancy, n(%) | | 237 (66.4%) | 137 (60.6%) | 0.157^a^ |
| Age, years | | 31 (29-34) | 31 (30-33) | 0.674^b^ |
| BMI, kg/m^2^ | | 23.0 (21.2-25.7) | 22.9 (21.2-25.4) | 0.583^b^ |
| Duration of infertility, years | | 3 (1-5) | 3 (1-4) | 0.525^b^ |
| Infertility, n(%) | Primary | 196 (54.9%) | 129 (57.1%) | 0.606^a^ |
|  | Secondary | 161 (45.1%) | 97 (42.9%) |  |
| Gravidity | 0 | 195 (54.6%) | 127 (56.2%) | 0.182^a^ |
|  | 1 | 86 (24.1%) | 60 (26.6%) |  |
|  | 2 | 56 (15.7%) | 22 (9.7%) |  |
|  | ≥3 | 20 (5.6%) | 17 (7.5%) |  |
| Parity | 0 | 308 (86.3%) | 193 (85.4%) | 0.610^#^ |
|  | 1 | 41 (11.5%) | 30 (13.3%) |  |
|  | ≥2 | 8 (2.2%) | 3 (1.3%) |  |
| History of abortion | No | 239 (66.9%) | 152 (67.3%) | 0.938^a^ |
|  | Yes | 118 (33.1%) | 74 (32.7%) |  |
| Surgical abortion | 0 | 312 (87.4%) | 189 (83.6%) | 0.128^a^ |
|  | 1 | 38 (10.6%) | 26 (11.5%) |  |
|  | ≥2 | 7 (2.0%) | 11 (4.9%) |  |
| Living children | 0 | 325 (91.0%) | 203 (89.8%) | 0.625^a^ |
|  | ≥1 | 32 (9.0%) | 23 (10.2%) |  |

^a^for chi-square test, and ^b^for Mann-Whitney U test. BMI: body mass index.
